# Cellulase and Xylanase from *Phanerochaete chrysosporium* ATCC24725: Production, Regulation of Synthesis, and Properties

**DOI:** 10.3390/biom16101446

**Published:** 2026-10-03

**Authors:** Eva Kachlishvili, Mikheil Asatiani, Aza Kobakhidze, Mariam Rusitashvili, Vladimir Elisashvili

**Affiliations:** Institute of Microbial Biotechnology, Agricultural University of Georgia, 240 David Aghmashenebeli Alley, 0159 Tbilisi, Georgia; e.kachlishvili@agruni.edu.ge (E.K.); m.asatiani@agruni.edu.ge (M.A.); a.kobakhidze@agruni.edu.ge (A.K.); mrusi2010@agruni.edu.ge (M.R.)

**Keywords:** *Phanerochaete chrysosporium*, cellulase, xylanase, lignocellulosic materials, submerged fermentation, regulation, properties

## Abstract

In this study, nine wood-rotting basidiomycete species were screened for cellulase and xylanase production in submerged fermentation of crystalline cellulose. *Phanerochaete chrysosporium* ATCC 24725 was the best producer of CMCase and xylanase, with enzymatic activity of 71.7 U/mL and 75.9 U/mL, respectively. The total cellulase, endoglucanase, and xylanase activities of *P. chrysosporium* ATCC24725 in the presence of lignocellulosic substrates were 3–7 times lower than the activity of these enzymes in the medium with crystalline cellulose. However, the endoglucanase activity of *Trametes ochracea* BCC76 in the medium containing tangerine pomace was the same as in the medium containing Avicel, whereas the xylanase activity was 3 times higher. Cellulase and xylanase synthesis in *P. chrysosporium* ATCC24725 occurs through induction mechanisms. Cellulose and cellulose-containing materials are the most suitable substrates for cellulase and xylanase production in *P. chrysosporium* ATCC24725, whereas CMC and xylan proved to be poor substrates for stimulating the secretion of these enzymes. In *P. chrysosporium* ATCC24725, cellulase and xylanase synthesis do not occur in the presence of easily metabolizable carbon sources due to catabolite repression, even if cellulose is present in the nutrient medium. Induction of enzyme synthesis resumes only after the easily metabolizable carbon source is depleted. The enzyme preparation isolated after culturing the fungus in a fermenter was partially purified and characterized. A crude cellulase preparation from *P. chrysosporium* ATCC24725 showed similar efficiency as Accellerase 1500 in the hydrolysis of alkali-pretreated wheat straw. The obtained results highlight the potential of *P. chrysosporium* ATCC 24725 as a reliable fungal cellulase producer for biorefinery applications.

## 1. Introduction

Microbial and enzymatic conversion of agricultural and food production waste into valuable resources based on the concept of lignocellulose biorefining is of ongoing interest to numerous research groups and companies worldwide [1,2]. Achieving these goals requires the selection of super-producers of lignocellulolytic enzymes, an understanding of the mechanisms that regulate the synthesis of cellulases and ligninases, and the establishment of the physicochemical and catalytic properties of these enzymes.

Basidiomycetes, which grow in nature on different types of plant material (living and dead trees, forest litter, grasses, decaying plant matter in soil), have very diverse abilities to degrade plant biomass by the expression of genes encoding lignocellulolytic enzymes [3,4,5]. The cellulase system consists of three major components, namely endoglucanase (EC 3.2.1.4), exoglucanase (EC 3.2.1.91), and β-glucosidase (EC 3.2.1.21), which act synergistically to convert cellulose into glucose [6,7]. Regarding hemicellulases, endo-β-1,4-xylanase (EC 3.2.1.8) and β-xylosidase (EC 3.2.1.37) are key enzymes required for the degradation of the xylan backbone. Cellulases and xylanases, which catalyze the hydrolysis of plant-based polysaccharides, are crucial enzymes in lignocellulose biorefineries.

Cellulases are synthesized by many microorganisms, although microbes belonging to the genera *Clostridium*, *Cellulomonas*, *Trichoderma*, *Aspergillus*, and other ascomycetes are considered the best cellulase producers. The production of cellulases and xylanases by fungi belonging to the Basidiomycota division has been understudied, even though these fungi are well adapted to degrading polysaccharides in lignified substrates and may be a promising source of enzymes for polysaccharide hydrolysis. Indeed, some basidiomycete fungi have shown exceptional potential to produce individual hydrolytic enzymes [8,9,10,11]. Thus, a very high yield of total cellulase activity (21.8 FPU/g) was obtained after solid-state fermentation (SSF) of banana pseudostem by *Pleurotus ostreatus* [12]. *Schizophyllum commune* BCC632 secreted 869 U/mL xylanase in submerged fermentation of 1.5% Avicel + 0.6% glycerol [13]. Remarkably, the cellulolytic activity exhibited by the litter-decomposing basidiomycete *Laetisaria arvalis* was 7.5 times higher than that of a reference *Trichoderma reesei* industrial strain [14]. Moreover, the enzyme preparations obtained from *Lentinus edodes* (1.785 mg/mg) and *Pleurotus ostreatus* (0.500 mg/mg) cultures exhibited higher activity in hydrolyzing carboxymethylcellulose than commercial cellulase (0.041 mg/mg) [15]. Furthermore, the thermostable endoglucanase from *Fomitopsis meliae* CFA 2 exhibited an exceptional half-life of 11.36 h at 70 °C. These results were achieved owing to both the discovery of effective enzyme producers and the elucidation of the role of individual nutrients, stimulators, and physicochemical factors in cellulase and xylanase synthesis [11,16,17].

The white-rot fungus *Phanerochaete chrysosporium* is a model microorganism with exceptional potential for synthesis of lignin-modifying enzymes and degradation of lignin [18,19]. The fungal genome contains more than 69 different families of carbohydrate-active enzymes, including 166 glycoside hydrolases (www.cazy.org). Surprisingly, there are many transcriptomic and proteomic studies to identify lignocellulose-degrading biocatalysts in *P. chrysosporium* during fermentation of lignocellulosic substrates, but information on the cellulase and xylanase activities of the fungus and the mechanisms regulating the synthesis of these enzymes is still limited. In particular, physiological factors modulating the activity of individual carbohydrate-active enzymes remain poorly understood, making it difficult to develop promising strategies for increasing the production of cellulases and xylanases. Furthermore, little is known about the properties of *P. chrysosporium* cellulase and xylanase and their applicability to lignocellulose saccharification in biorefineries.

This study aimed to evaluate the cellulase and xylanase activity of taxonomically and ecologically diverse wood-destroying basidiomycetes during submerged fermentation of crystalline cellulose and a range of lignocellulosic materials, as well as to establish, for the first time, the main features of the regulation of synthesis and properties of cellulase and xylanase in *P. chrysosporium* ATCC24725.

## 2. Materials and Methods

### 2.1. Organisms and Inoculum Preparation

*Coprinus comatus* BCC801, *Fomes fomentarius* BCC38, *Irpex lacteus* BCC104, *Panus lecomtei* BCC903, *Phanerochaete chrysosporium* ATCC24725, *Piptoporus betulinus* BCC520, *Pleurotus ostreatus* BCC518, *Trametes gibbosa* BCC22, and *Trametes ochracea* BCC76 from the Institute Basidiomycetes culture collection were used in this study. The fungal inoculum was prepared by growing five mycelial agar plugs on a rotary shaker at 160 rpm and 27 °C in 250 mL flasks containing 100 mL of standard medium (g/L): glucose—15.0, KH_2_PO_4_—0.8, K_2_HPO_4_—0.6, MgSO_4_ × H_2_O—0.5, peptone—3.0, yeast extract—3.0, and pH—6.0. After 7 days of fungal cultivation, mycelial pellets were harvested and homogenized using a Waring laboratory blender.

### 2.2. Cultivation Conditions

The submerged cultivation of the fungi was carried out using a rotary shaker Innova 44 at 160 rpm and 27 °C in 250 mL flasks containing 100 mL of a medium of the following composition (g/L): crystalline cellulose (Avicel)—15.0, KH_2_PO_4_—0.8, K_2_HPO_4_—0.6, MgSO_4_ × 7H_2_O—0.5, peptone—3.0, yeast extract—3.0, and pH—5.8. To study the effect of lignocellulosic growth substrates on fungal enzyme activity, the following materials were added to the media as the only carbon sources: Avicel and cellulose isolated from tangerine peels at a concentration of 1%, as well as mandarin pomace, wheat bran, wheat straw, and vinasse from corn at a concentration of 4%. Preliminarily, lignocellulosic materials were dried in an oven at 50 °C and milled into powder. Before sterilization, the media containing plant materials were boiled and cooled, and the pH was adjusted to 6.0. To study the enzyme activity of *P. chrysosporium* ATCC24725 as a function of carbon source concentration, crystalline cellulose (Avicel), soluble carboxymethyl cellulose (CMC), and xylan were added to the medium at 0.5%, 1%, and 2%. Each experiment was performed twice in triplicate at each time point. During the fungus cultivation, at predetermined time intervals, 1–2 mL samples were taken from the flasks. The solids were separated by centrifugation at 10,000× *g* for 10 min at 4 °C, and the supernatants were analyzed for pH, reducing sugars, and enzyme activities.

In experiments to establish the induction of cellulase and xylanase synthesis, *P. chrysosporium* ATCC24725 was grown in four media containing (1) 0.5% glycerol, (2) 1.5% Avicel, (3) 0.5% glycerol + 1.5% Avicel, and (4) 0.5% glycerol + 1.5% Avicel added on day 3. To study the catabolite repression of cellulase and xylanase synthesis, the fungus was cultivated for 4 days in a nutrient medium containing 1.5% Avicel, then 0.3% glucose, 0.6% glucose, or 0.6% glycerol was added to the culture. To monitor the time course of enzyme accumulation, 1 mL samples were taken from flasks at the time of inoculation and at 24-h intervals, and the solids were separated by centrifugation at 10,000× *g* for 10 min at 4 °C.

### 2.3. Cultivation in a Fermenter

To scale up the cellulase and xylanase production, *P. chrysosporium* ATCC24725 was cultivated in a 7 L fermenter LiFlus GX (Biotron, Bucheon, Republic of Korea) equipped with three Rushton impellers and pH, temperature, and pO_2_ probes. In this case, the fermenter was filled with 5 L of the medium containing (g/L) Avicel—20.0 or Avicel (15.0) + tangerine pomace (25.0), KH_2_PO_4_—0.8, K_2_HPO_4_—0.6, MgSO_4_—0.5, yeast extract—3.0, and peptone—3.0. Polypropylene glycol 2000 (3 mL) was added as an antifoam agent. The fermenter was sterilized at 121 °C for 40 min and inoculated with homogenized fungal mycelium. Fermentation was carried out without baffles at a temperature of 27 °C, with a constant air flow rate of 1 *v*/*v*/min. The agitation rate was gradually increased from 150 to 300 rpm. During the fermentation process, samples were collected daily and analyzed for enzyme activity. After fermentation, the fungal biomass was separated from the culture liquid by successive filtration and centrifugation at 6000 rpm for 20 min at 4 °C.

### 2.4. Isolation and Characterization of Cellulase and Xylanase from P. chrysosporium ATCC24725

After fermentation and biomass separation, the culture liquid was concentrated by precipitation with ammonium sulfate (75% saturation). The protein precipitate was collected by centrifugation, dissolved in 50 mM citrate buffer (pH 6), and desalted by gel-permeation chromatography using a Sephadex G-10 column equilibrated with the same buffer. The active fractions, containing cellulase and xylanase activities, were pooled and characterized.

The optimum pH for endoglucanase and xylanase activities was determined by incubating the obtained enzyme preparation with 1% CMC or xylan using a 0.1 M citrate–phosphate buffer with pH values from 4.0 to 7.5. CMCase and xylanase pH stability was studied by pre-incubating the enzyme preparation at different pH levels at 27 °C for 24 h, followed by the enzyme activity assay. The optimal working temperature for endoglucanase and xylanase activity was determined by incubating the enzyme preparation under standard conditions with 1% CMC or xylan at various temperatures (20–70 °C). Thermostability of CMCase and xylanase was determined by pre-incubating the enzyme preparation for 24 h at temperatures from 30 to 70 °C, followed by measuring the remaining enzyme activity. To evaluate the substrate specificity of endoglucanase and xylanase and K_m_, enzyme activities were measured under standard assay conditions in reaction mixtures containing 0.05–2% birchwood xylan or CMC.

### 2.5. Wheat Straw Saccharification

Enzymatic hydrolysis of the wheat straw was performed under standard conditions (0.1 mM citrate buffer, pH 5.0, 50 °C) with gentle agitation at 150 rpm for 48 h. Base-pretreated (1.5% NaOH) wheat straw with a composition of 65% cellulose, 19% hemicellulose, and 9% lignin was used as the substrate. Accellerase 1500 (Genencor, Finland) and crude enzyme preparation isolated from *P. chrysosporium* ATCC24725 cultivated in a fermenter were used as enzyme sources. The reaction mixture (10 mL) contained 200 mg of substrate, 10 or 20 FPU/g substrate, and 300 µg tetracycline to prevent microbial contamination. Samples were taken from the reaction mixtures after 24 and 48 h of saccharification, heated in a boiling water bath for 2 min, and then centrifuged at 10,000× *g* for 5 min at 4 °C. The supernatants were analyzed for reducing sugars using the dinitrosalicylic acid reagent method [20].

### 2.6. Analytical Methods

The total cellulase activity (filter paper activity, FPA) was measured using Whatman filter paper No. 1 according to IUPAC recommendations [21]. Endoglucanase (CMCase) activity was assayed according to the IUPAC recommendations using 1% low-viscosity carboxymethyl cellulose in 50 mM citrate buffer (pH 5.0) at 50 °C for ten minutes [21]. Xylanase activity was determined using 1% birchwood xylan (Roth 7500) in the same citrate buffer at 50 °C for 10 min [22]. Glucose and xylose standard curves were used to calculate the cellulase and xylanase activities. In all assays, the release of reducing sugars was measured using the dinitrosalicylic acid reagent method. One unit of enzyme activity was defined as the amount of enzyme releasing 1 μmoL of reducing sugars per minute.

### 2.7. Statistical Analyses

All experimental data are presented as mean ± standard deviation (SD). Statistical differences among experimental groups were evaluated using one-way analysis of variance (ANOVA), followed by Tukey’s (HSD) post hoc test for multiple comparisons. For experiments involving different fungal strains, growth substrates, or polysaccharide concentrations, the statistical analysis was performed separately for each measured response variable. For wheat straw saccharification experiments, comparisons among enzyme treatments were performed separately at each hydrolysis time (24 and 48 h). Differences were considered statistically significant at *p* < 0.05. In the tables, different superscript lowercase letters within the same column indicate statistically significant differences, whereas values sharing at least one common letter are not significantly different.

## 3. Results

### 3.1. Screening of Basidiomycetes for Cellulase and Xylanase Activities

The ability of one brown-rot and eight white-rot fungi to secrete cellulase and xylanase during submerged fermentation of crystalline cellulose was assessed. Visually, all fungi grew well in the presence of Avicel in the form of small pellets (2–4 mm). Of these, *C. comatus* BCC801, *P. betulinus* BCC520, and *P. ostreatus* BCC518 were distinguished by an increase in the pH of the medium by the end of cultivation to values from 8.2 to 8.5. *P. chrysosporium* ATCC24725 appeared to be the best producer of CMCase activity, with 71.7 U/mL after 10 days of fungal cultivation (Table 1). *I. lacteus* BCC104, followed by *T. gibbosa* BCC22, was also found to be a good producer of endoglucanase. The remaining fungal cultures showed many times lower CMCase activity. Measurement of total cellulase activity revealed significant filter paper hydrolysis potential in cultures of *I. lacteus* BCC104 and *P. chrysosporium* ATCC24725. It is interesting to note that *F. fomentarius* BCC38, despite having a relatively low CMCase activity (4.2 U/mL), exhibited a fairly high FPA (0.83 U/mL). The xylanase activity of the tested fungi varied from 0.8 U/mL in *P. betulinus* BCC520 to 75.9 U/mL in *P. chrysosporium* ATCC24725. *I. lacteus* BCC104, followed by *T. gibbosa* BCC22, also expressed significant xylanase activity.

### 3.2. Effect of Lignocellulosic Growth Substrates on Basidiomycetes Enzyme Activity

To determine the influence of lignocellulosic materials with different chemical compositions on cellulase production, three fungal strains were selected. Cellulase and xylanase activities of fungi during submerged fermentation of tangerine pomace, vinasse from corn, wheat bran, and wheat straw were compared with those during fungal cultivation in the presence of Avicel. Visual observations revealed rapid and abundant fungal growth in the form of small pellets from the first days of cultivation in the presence of pomace, wheat bran, and vinasse. Wheat straw was a relatively poor substrate, although significant mycelial biomass was observed toward the end of fungal cultivation. In all media, fungal growth was accompanied by an increase in medium pH from 6.0 to 6.2–7.6 towards the end of cultivation (Table 2).

The tested fungi exhibited comparatively high cellulase and xylanase activities regardless of the material tested; however, enzyme yields varied significantly. Among the five growth substrates tested, crystalline cellulose was the best carbon source, providing the highest enzyme activity of all fungi (Table 2). *P. chrysosporium* ATCC24725 appeared to be an outstanding producer of cellulase and xylanase in the presence of crystalline cellulose and had the highest endoglucanase (72.1 U/mL), FPA (2.51 U/mL), and xylanase (84.6 U/mL) activities; other materials provided 3–7-fold lower CMCase activity of the fungal culture. Similarly, the total cellulase and xylanase activities of *P. chrysosporium* ATCC24725 in the presence of lignocellulosic substrates were approximately 5 and 3–4 times lower, respectively, than the activity of these enzymes in the medium with crystalline cellulose.

In two other fungal cultures, a somewhat different response to the presence of chemically and structurally different lignocellulosic substrates was revealed. In particular, the endoglucanase activity of *T. ochracea* BCC76 in the medium containing tangerine pomace was the same as in the medium containing Avicel, whereas the xylanase activity was 3 times higher. Wheat straw also ensured a comparatively high endoglucanase activity of the fungal culture compared to Avicel, whereas the straw and vinasse provided the same xylanase activity. It should also be noted that *T. gibbosa* BCC22 exhibited high total cellulase activity in the presence of plant substrates. However, the xylanase activity of this culture was quite low compared to the cellulose-containing medium (Table 2).

Analysis of profiles of enzyme activity accumulation showed that they depend both on the fungal species and the type of growth substrate. In all cultures, the CMCase activity of the fungi gradually increased and achieved a maximum after 10–14 days of cultivation, while the FPA of *T. gibbosa* BCC22 and *T. ochracea* BCC76 peaked earlier, after 4–7 days of cultivation. It is worth noting that in *P. chrysosporium* ATCC24725 cultures grown in the presence of Avicel, tangerine pomace, and alcohol production waste, the maximum concentration of FPA, compared to media containing wheat bran and wheat straw, was also achieved earlier, after 4–7 days. The xylanase activity of the three fungal cultures increased rapidly only during 7 days of growth in a medium containing crystalline cellulose. In the other media, the highest xylanase activity of the fungi was observed after 10–14 days of fermentation.

### 3.3. Effect of Polysaccharide Carbon Sources on Cellulase and Xylanase Activities of P. chrysosporium ATCC24725

Based on the data received, *P. chrysosporium* ATCC24725 was selected for subsequent experiments. To elucidate the features of cellulase and xylanase production by *P. chrysosporium* ATCC24725 in the presence of polymeric carbon sources and potential inducers of enzyme synthesis, the influence of cellulose and xylan concentrations on these processes was examined. In the control medium without polysaccharides, only peptone and yeast extract could serve as a carbon source for fungal growth. The results showed that the water-soluble glucuronoxylan extracted from *Betula alba* (birch wood, Carl Roth) was a poor growth substrate and an inducer of hydrolase synthesis. A cellulose derivative, carboxymethyl cellulose (CMC), supported detectable fungal growth and the production of both cellulases and xylanases (Table 3). However, the enzymatic activity of the fungal culture was many times lower than in a medium with crystalline cellulose. Further, the results show that the cellulase and xylanase activities of *P. chrysosporium* ATCC24725 are significantly dependent on the cellulose concentration in the culture medium. This is especially true for the production of endoglucanase and xylanase. Thus, increasing the concentration of Avicel from 0.5 to 1% more than doubled the endoglucanase activity of *P. chrysosporium* ATCC24725 and almost tripled the xylanase activity of the culture. The Avicel concentration of 2% was sufficient to obtain the highest yield of target enzymes. Varying the concentration of carboxymethyl cellulose from 0.5 to 1% had a lesser effect on the production of CMCase, but doubled the activity of xylanase of *P. chrysosporium* ATCC24725. Increasing the concentration of soluble cellulose to 2% resulted in a slight increase in the yields of endoglucanase and xylanase. Finally, compared to other enzymes, the total cellulase activity was less dependent on the polysaccharide concentrations in the nutrient medium.

### 3.4. Induction and Catabolite Repression of Cellulase Synthesis in P. chrysosporium ATCC24725

**Figure 1:**
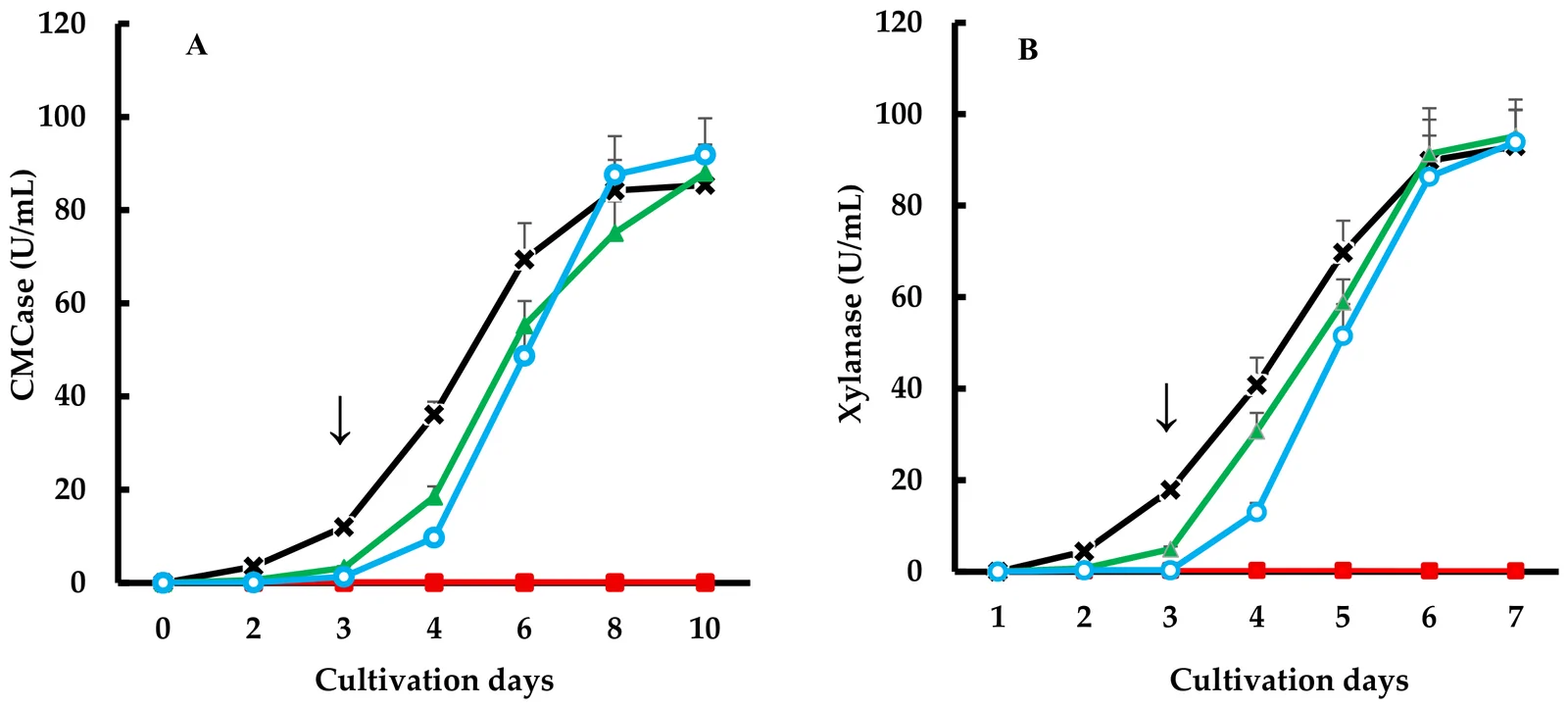
Induction of *P. chrysosporium* ATCC24725 endoglucanase (**A**) and xylanase (**B**) synthesis. The fungi were grown in media containing 1.5% Avicel (x), 0.5% glycerol (■), 0.5% glycerol + 1.5% Avicel (▲), and 0.5% glycerol + 1.5% Avicel on day 3 (○). The arrows indicate the time at which cellulose was added to the glycerol-containing medium.

In a subsequent set of experiments, the mechanisms regulating cellulase and xylanase synthesis by *P. chrysosporium* ATCC24725 were studied. The previous results indicate that these enzymes are produced only under conditions in which the fungus uses cellulosic materials as a growth substrate. Glycerol was used as a non-inducing carbon source, ensuring good growth of *P. chrysosporium* ATCC24725 and a constitutive level of hydrolases. Indeed, only traces of cellulase and xylanase could be detected at different stages of *P. chrysosporium* ATCC24725 growth in medium containing 5 g/L glycerol (Figure 1). In Avicel-containing medium, the cellulase and xylanase activities were detected from the second day of cultivation, and they gradually increased till the end of the experiments, achieving 85.4 and 93.0 U/mL, respectively. When the 0.5% glycerol-containing culture was supplemented with the crystalline cellulose from the beginning of cultivation, a delay in cellulase and xylanase formation was observed during two days of *P. chrysosporium* ATCC24725 cultivation. Subsequently, the synthesis and secretion of both enzymes occurred very rapidly. Moreover, the CMCase activity of *P. chrysosporium* ATCC24725 by the end of cultivation in this medium reached the same level as in the medium containing Avicel, whereas the xylanase activity of the fungus exceeded that in the control medium containing only cellulose. When Avicel was added to the *P. chrysosporium* ATCC24725 culture growing in the presence of 0.5% glycerol for 3 days, secretion of cellulase and xylanase began within 24 hours; on the 10th day of cultivation, the xylanase activity of the fungus was comparable to the enzyme activity observed in the cellulose-based medium, while the endoglucanase activity slightly exceeded that in the medium with Avicel.

**Figure 2:**
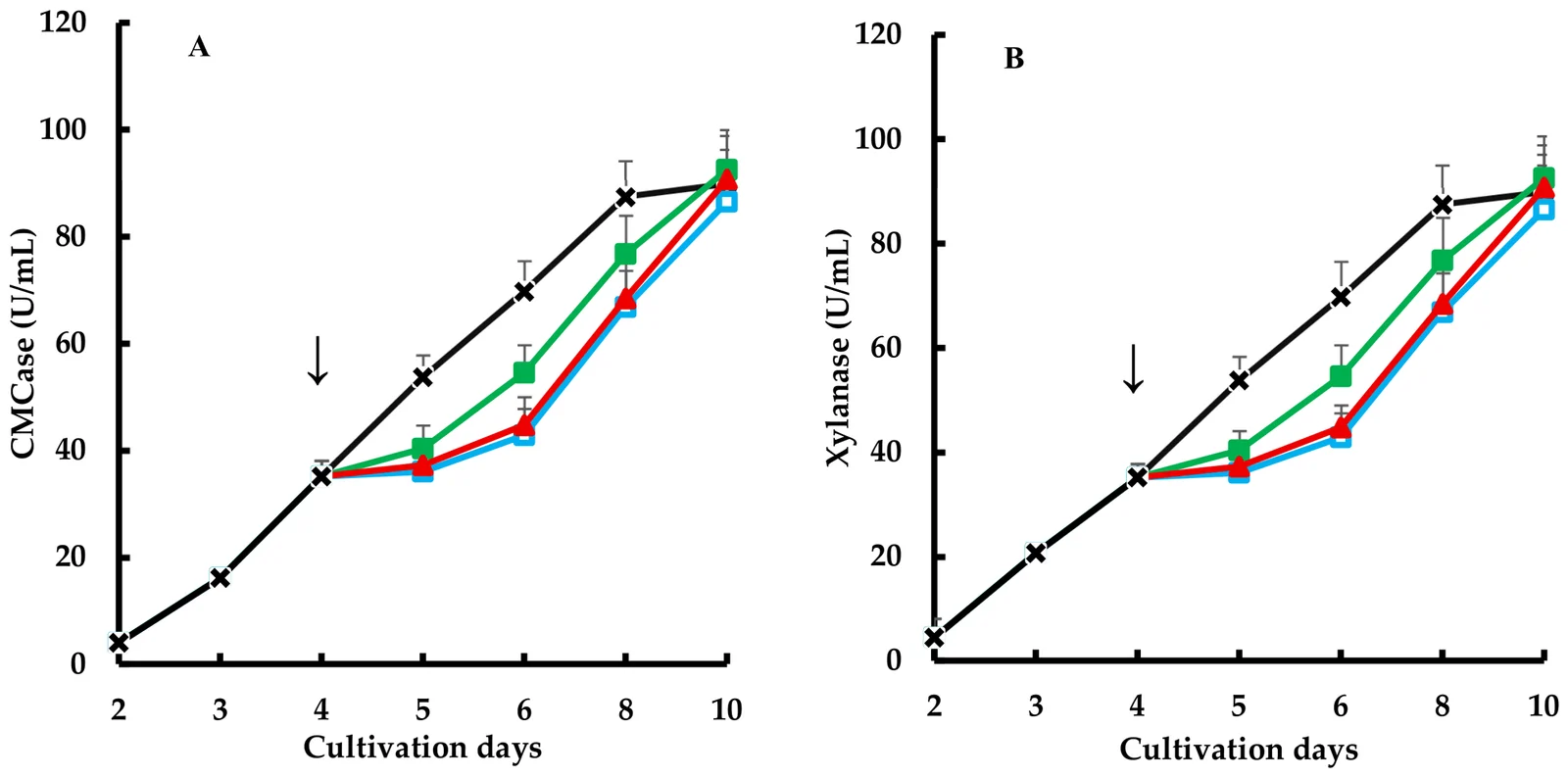
Catabolite repression of *P. chrysosporium* ATCC24725 endoglucanase (**A**) and xylanase (**B**) synthesis. In the control, the fungi were grown in medium containing 1.5% Avicel (x). To induce catabolite repression, 0.3% glucose (■), 0.6% glucose (□), and 0.6% glycerol (▲) were added to cultures grown in the presence of 1.5% Avicel for 4 days. The arrows indicate the time at which glucose or glycerol was added to the cellulose-containing medium.

To study the catabolite repression of cellulase and xylanase synthesis in *P. chrysosporium* ATCC24725, 0.3% and 0.6% glucose, as well as 0.6% glycerol at final concentrations, were added to cultures grown in the presence of 1.5% Avicel for 4 days. In the control, where a cellulose-based medium was used, the enzyme activities gradually increased and achieved a maximum by day 10 (Figure 2). When glucose or glycerol was added to the medium containing Avicel, the production of CMCase and xylanase ceased, even though the inducer was still present in the medium. It should be noted that the higher the glucose concentration in the medium, the longer the period of catabolite repression of cellulase and xylanase synthesis. However, after a short delay, enzyme secretion resumed, confirming the reversibility of the mechanism of hydrolase synthesis repression by easily metabolized carbohydrates. Interestingly, the rates of CMCase and xylanase accumulation after depletion of glycerol or glucose were somewhat higher than those in Avicel-containing culture. As a result, after 10 days of submerged cultivation of *P. chrysosporium* ATCC24725 in a medium with two carbon sources, the volumetric enzymatic activities of the fungus reached or slightly exceeded those in the Avicel-containing medium.

### 3.5. Scaled-Up Production of Cellulase and Xylanase by P. chrysosporium ATCC24725 in a Fermenter

In the final stage, the feasibility of obtaining cellulase and xylanase at high yield was tested by cultivating *P. chrysosporium* ATCC24725 in a fermenter. In the fermentation process, the medium pH was maintained for three days at 6.0 to provide optimal conditions for fungal growth; then it was changed to pH 5.0 to ensure optimal conditions for cellulose hydrolysis. After five days, the pH of the medium was maintained at 6.2 to slow down cellulose saccharification and prevent sugar accumulation and, consequently, catabolite repression of *P. chrysosporium* ATCC24725 enzyme synthesis.

**Figure 3:**
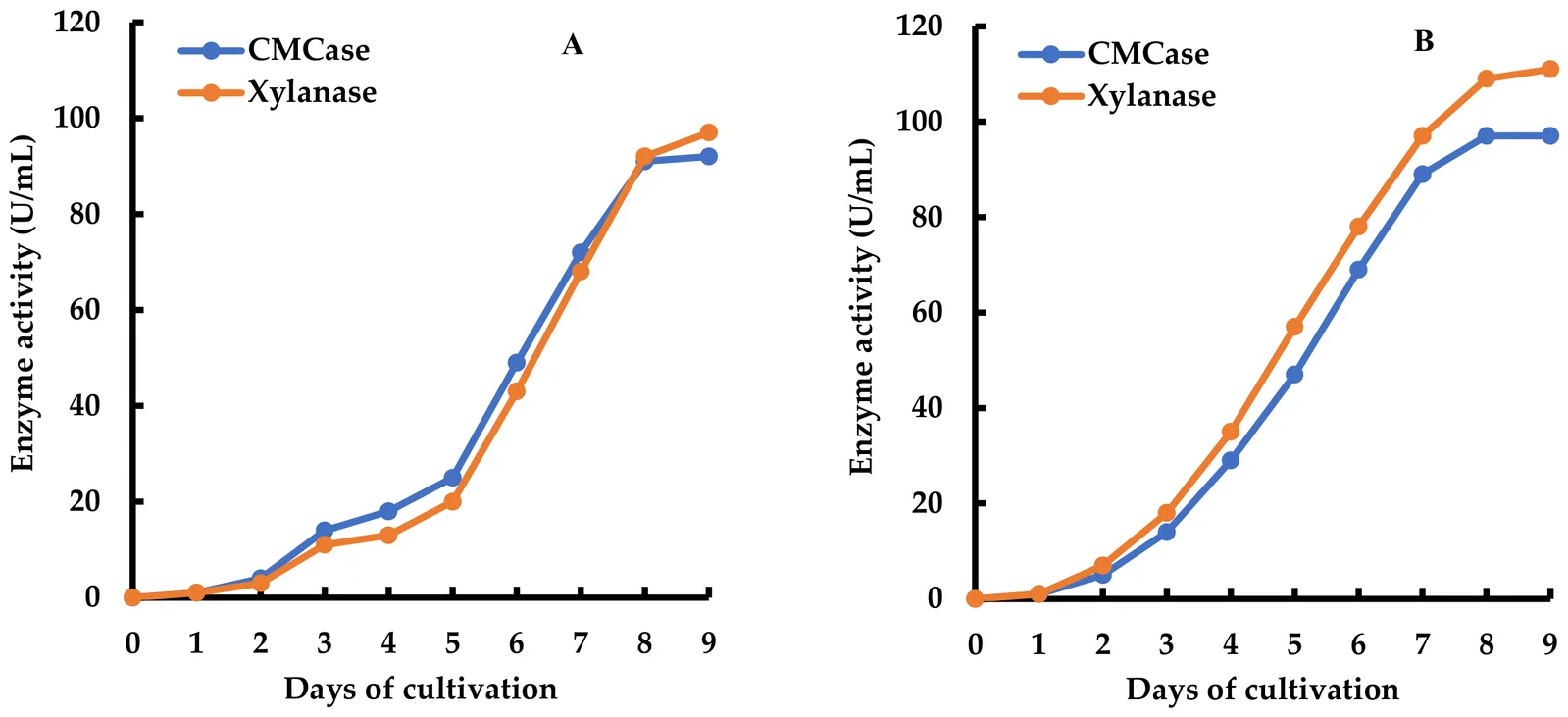
Kinetics of CMCase and xylanase activity accumulation in *P. chrysosporium* ATCC24725 cultivation in a laboratory fermenter containing media with 20 g/L Avicel (**A**) or 15 g/L Avicel + 25 g/L mandarin pomace (**B**) as carbon sources.

The results presented in Figure 3A show that cellulase and xylanase activities appeared after one day of fermentation and rapidly increased synchronously during the first three days of fermentation. Then, over the course of two days, the rates of accumulation of extracellular cellulase and xylanase activities decreased somewhat, possibly due to partial inactivation of the synthesized enzymes at pH 5 or due to the appearance of sugars as a result of cellulose hydrolysis. However, during subsequent fermentation, the CMCase and xylanase activities gradually increased, reaching maximums of 92 and 97 U/mL, respectively, on days 8–9.

It should be noted that during the first days of *P. chrysosporium* ATCC24725 cultivation, the accumulation of enzymatic activity occurred comparatively slowly. We hypothesized that the main reason for this was the slow growth of the fungus due to the lack of an easily metabolizable carbon source. Therefore, in the next fermentation experiment, *P. chrysosporium* ATCC24725 was cultivated in a medium containing, in addition to Avicel (15 g/L), tangerine pomace (25 g/L) to ensure a rapid initial growth of the fungus. Moreover, the fermentation was carried out at a constant medium pH of 5.5, which favors both the hydrolysis of polysaccharides and secretion of the target enzymes. The fermentation results presented in Figure 3B show that, in this case, secretion of both enzymes began on the second day of cultivation, and cellulase and xylanase activities increased continuously, reaching a maximum of 97 and 111 U/mL, respectively, on day 8. Thus, the addition of tangerine pulp to the nutrient medium clearly accelerates the fungus’s initial growth and the production of hydrolases, especially xylanase, and ultimately results in a slight increase in the yield of target enzymes.

### 3.6. Characterization of Cellulase and Xylanase from P. chrysosporium ATCC24725

**Figure 4:**
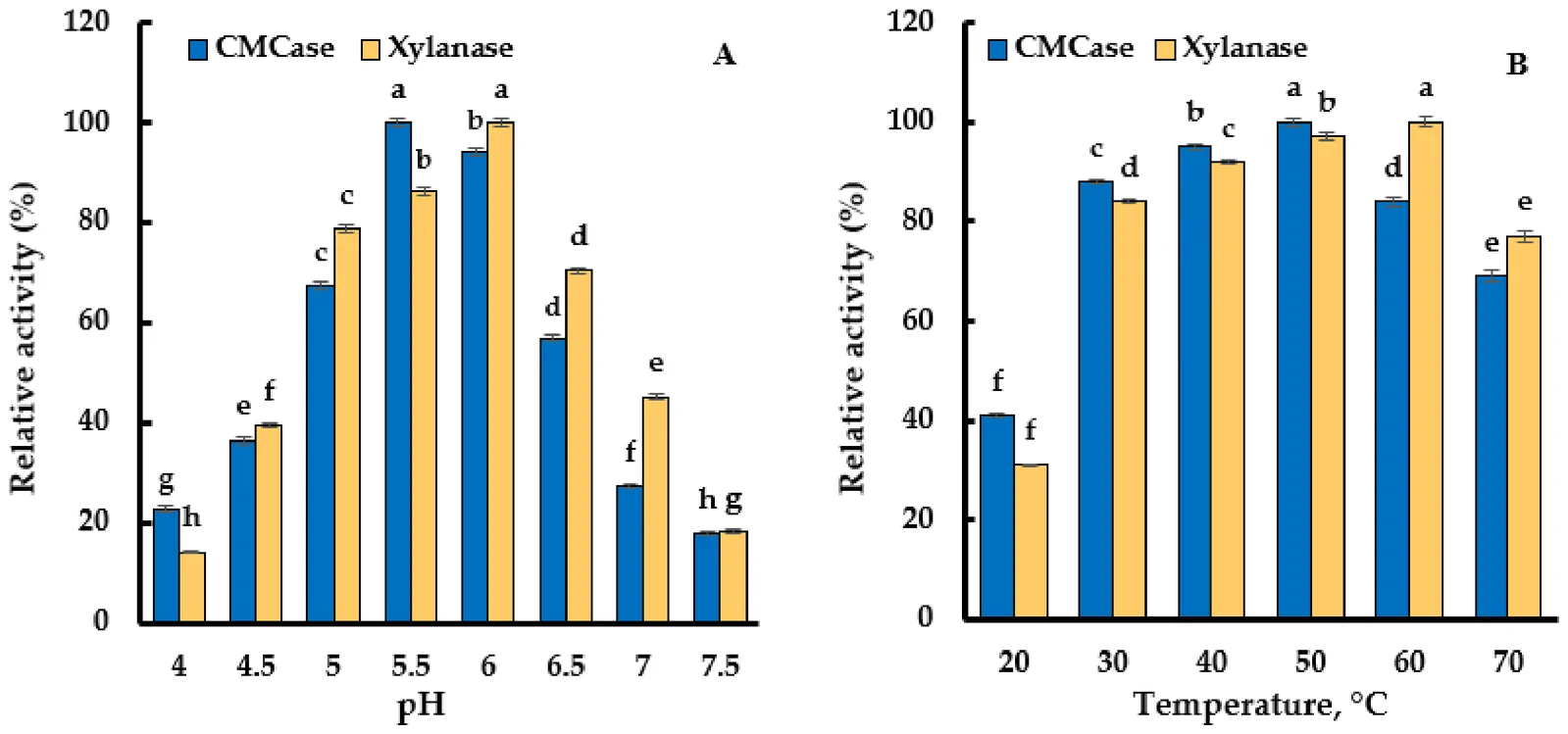
Dependence of CMCase and xylanase activity of *P. chrysosporium* ATCC24725 on pH (**A**) and temperature (**B**) of the reaction mixture.

The key properties of CMCase and xylanase of the preparation obtained after cultivation of *P. chrysosporium* ATCC24725 in a fermenter were investigated. When studying the effect of reaction mixture pH on endoglucanase and xylanase activity, it was noted that with an increase in pH from 4 to 5.5, a gradual increase in the activity of both enzymes was observed (Figure 4A). Then, with an increase in pH to 6, CMCase activity decreased slightly, whereas xylanase activity reached its maximum. With a further increase in pH to 6.5 and above, a sharp decrease in the activity of both enzymes was observed. Nevertheless, CMCase and xylanase still retained approximately 18% of their maximum activity at pH 7.5. Thus, the optimal pH for endoglucanase and xylanase activities was 5.5 and 6.0, respectively, but both enzymes exhibited hydrolytic activity over a wide pH range.

The effect of the reaction mixture temperature on *P. chrysosporium* ATCC24725 CMCase and xylanase activities was evaluated in the range of 20–70 °C. The study showed that both enzyme activities increased approximately twofold as the temperature of incubation was elevated from 20 to 30 °C (Figure 4B). The maximum activity of *P. chrysosporium* ATCC24725 CMCase was detected at a temperature of 50 °C, and xylanase activity at 60 °C. Both enzymes showed high hydrolytic activity in the reaction mixture at 70 °C; specifically, endoglucanase and xylanase activities were reduced by 31% and 23%, respectively.

**Figure 5:**
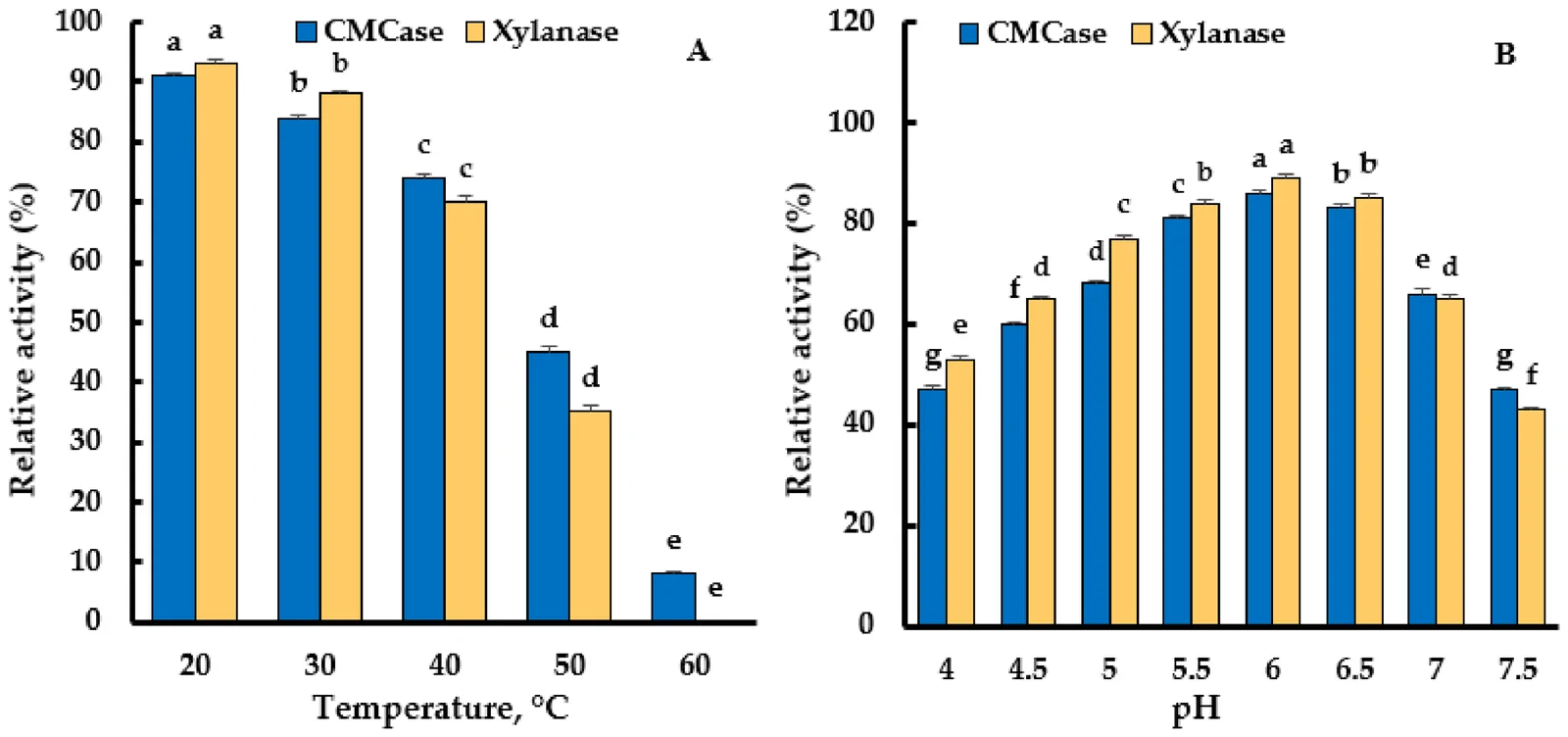
Thermostability (**A**) and pH stability (**B**) of CMCase and xylanase from *P. chrysosporium* ATCC24725. The residual activity of enzymes after 24 hours of incubation under the specified conditions is expressed as a percentage of their initial activity.

The study of pH stability and thermostability of *P. chrysosporium* ATCC24725 enzymes showed that CMCase and xylanase are quite stable at room temperature, retaining, respectively, 91 and 93% of the initial activity after 24 h of incubation at 20 °C and 84 and 88% at 30 °C (Figure 5A). However, when incubated for the same time at 50 °C, cellulase and xylanase lost 55 and 65%, respectively, of their initial activity. No xylanase activity and 8% residual CMCase activity were detected after 24 h of enzyme preparation incubation at 60 °C; i.e., the CMCase activity of the fungus at elevated temperatures is more stable than the xylanase activity. Minor differences between the endoglucanase and xylanase activities were detected when the enzyme preparation from *P. chrysosporium* ATCC24725 was incubated at different pH. Both enzymes exhibited the greatest stability when incubated for 24 h at pH 6 (Figure 5B). A decrease or increase in pH by one unit from the optimum resulted in a loss of 32 and 34% of the initial CMCase activity and 23 and 35% of xylanase activity, respectively. This means that, compared to xylanase, the endoglucanase activity of *P. chrysosporium* ATCC24725 is more sensitive to acidic conditions but more stable in neutral and slightly alkaline media.

Finally, the study of the effect of substrate concentration on the *P. chrysosporium* ATCC24725 endoglucanase and xylanase activities revealed that the Michaelis–Menten constants K_m_ calculated using the Lineweaver–Burk plot are 5.7 g/L (in the presence of carboxymethyl cellulose) and 5.3 g/L (in the presence of xylan) for CMCase and xylanase, respectively.

### 3.7. Saccharification of Wheat Straw by P. chrysosporium ATCC24725 and Commercial Cellulases

In this study, the hydrolysis of pretreated wheat straw (WS) was performed using a commercial enzyme and the crude cellulase preparation obtained after the fungus cultivation in a fermenter. At the Accellerase 1500 and *P. chrysosporium* ATCC24725 cellulase loads of 10 U FPA/g substrate, WS saccharification during 24 h hydrolysis resulted in total reducing sugar yields of 5 mg/mL and 5.3 mg/mL, respectively. Hydrolysis for 48 h increased the total reducing sugar content to 6.4 mg/mL and 6.7 mg/mL, respectively (Table 4). The slight increase in the yield of reducing sugars under the action of the cellulase from *P. chrysosporium* ATCC24725 compared to the commercial enzyme can be explained by the fact that the obtained enzyme preparation contains a complex of cellulases and hemicellulases involved in the hydrolysis of all WS polysaccharides. Doubling the concentrations of the commercial and in-house enzymes accelerated the hydrolysis rate and increased the reducing sugar content to 7.6 mg/mL and 7.8 mg/mL, respectively, after 48 h of incubation. These concentrations correspond to a sugar yield from wheat straw of 53% of the theoretically possible level.

## 4. Discussion

The production of cellulases and xylanases has been studied in numerous basidiomycete species across different taxonomic and ecological groups. These studies revealed significant interspecific differences in the ability of basidiomycetes to secrete cellulase and xylanase activities. In particular, the CMCase activity varied from 0.2 U/mL in cultures of *Coniophora puteana* [23] and *Pycnoporus sanguineus* [24] to 122 U/mL in *Pseudotrametes gibbosa* [25]. Remarkably, some basidiomycete strains expressed exclusively high enzymatic activities under appropriate culture conditions. Thus, *Armillaria gemina* secreted 146 U/mL endoglucanase, 15 U/mL ß-glucosidase, and 1.72 U/mL FPA [8], while *Schizophyllum commune* produced 740 U/mL xylanase and 18.6 U/mL ß-glucosidase [10]. In this study, basidiomycete fungi were screened to identify cellulase and xylanase producers with high catalytic efficiency on insoluble cellulose-containing substrates for use in biorefineries. Among the tested fungi, low endoglucanase activity (0.2 U/mL) was detected in the brown rot fungus *P. betulinus* BCC520 and white rot fungus *P. ostreatus* BCC518 (1.8 U/mL), while the highest enzyme activity (71.7 U/mL) was revealed in *P. chrysosporium* ATCC24725. The xylanase activity of the tested fungi varied from 0.8 U/mL in *P. betulinus* BCC520 to 75.9 U/mL in *P. chrysosporium* ATCC24725. These cellulase and xylanase activities of *P. chrysosporium* ATCC24725 are higher than previously obtained yields for the majority of basidiomycete species, including *P. chrysosporium* [11,26,27,28,29,30].

Many studies have examined the use of agro-industrial lignocellulosic residues as an inexpensive raw material for producing cellulolytic enzymes [7,11,31,32,33]. Consistent with these studies, the results presented in this paper indicate that cellulose and natural lignocellulosic materials significantly affected cellulase and xylanase expression in the basidiomycetes studied. It is worth noting that crystalline cellulose was the best carbon source, providing the highest enzyme activity of the three fungal species. In particular, the activities of CMCase, FPA, and xylanase of *P. chrysosporium* ATCC24725 were 3–6 times higher in a medium containing Avicel, compared to the activity of the enzymes when the fungus was grown in the presence of plant residues. Nevertheless, the presence of tangerine pomace in the nutrient medium provided the same endoglucanase activity as Avicel for *T. ochracea* BCC76, while xylanase activity was 3 times higher. It can be assumed that the activity of cellulase and xylanase in fungi depends not only on their physiological peculiarities and biosynthetic potential, but also on the composition and properties of their enzymatic systems, as well as on the ability of lignocellulosic materials to provide the fungal culture with both a carbon source and inducers of cellulase and xylanase synthesis. Consequently, the availability of various specific inducers of cellulase and/or hemicellulase expression in individual lignocellulosic materials makes it possible to select the necessary growth substrate for the production of enzyme cocktails with different sets and ratios of enzymes for various purposes.

*P. chrysosporium* has served as a model organism for the study of the fungal ligninolytic system and is the best-studied basidiomycete. Although a variety of biochemical and genetic tools and approaches have been used to investigate the function of *P. chrysosporium* genes responsible for lignocellulose degradation [34,35], the physiological aspects modulating the activity of individual carbohydrate-active enzymes remain poorly understood, which makes it difficult to develop promising strategies for increasing the production of cellulases and xylanases. It should be noted that most earlier investigations were based on the use of complex plant materials as carbon sources, with only a few studies using pure inducers. In this study, chemically and structurally different polysaccharides (crystalline cellulose, water-soluble carboxymethyl cellulose, and xylan in concentrations of 0.5–2%) were used as carbon sources for *P. chrysosporium* ATCC24725 cultivation to evaluate their effects on glycoside hydrolase production. In addition to the fact that Avicel provides the highest yields of cellulase and xylanase in submerged cultivation of *P. chrysosporium* ATCC24725, the choice of polysaccharides is explained by the fact that only a few specific enzymes are required for their hydrolysis. In contrast, the degradation of lignocellulosic materials requires the participation of a wide range of hydrolases and oxidases, the synthesis of which requires significant energy and material resources.

Experiments have shown that in the absence of polysaccharides, *P. chrysosporium* ATCC24725 does not produce cellulase and xylanase. However, among the tested polysaccharides, only crystalline cellulose provided intensive synthesis of glycoside hydrolases. Replacing Avicel in the nutrient medium with CMC significantly reduced the enzyme yield in the fungal culture, whereas in the presence of xylan, only trace cellulase activity and very low xylanase activity were detected. Similarly, the highest xylanase activity was produced by the same strain of *P. chrysosporium* in Avicel PH102-containing medium (38 U/mL), whereas the xylan-based medium resulted in the lowest enzyme activity (0.02 U/mL) [26]. The authors attributed the low xylanase activity to the rapid degradation of xylan and the inhibitory effect of the end products of hydrolysis—xylose and xylobiose. Specifically, 5.8 mg/mL of xylose was detected in a 6-day xylan-based culture, while the glucose content in the cellulose-containing medium was 0.05 mg/mL.

In our experiments, when cultivating *P. chrysosporium* ATCC24725 in media with Avicel and 0.5% CMC, no reducing sugars were detected throughout the entire cultivation period. Probably due to this, the production of cellulase and xylanase continued until the end of the fungal cultivation. In contrast, the concentration of reducing sugars was high in the 4-day fungal culture in medium with 2% xylan (3.7 mg/mL), and the low activity of glycoside hydrolase can be explained by the presence of catabolite repression of their synthesis. However, when culturing the fungus in media containing 1% and 2% CMC and 0.5% and 1% xylan, the concentration of reducing sugars in the culture fluids did not exceed 0.2 mg/mL. Nevertheless, the extracellular activity of cellulase and xylanase was low. Perhaps this phenomenon can be explained by the fact that, in media with CMC and xylan, *P. chrysosporium* ATCC24725 secretes very low β-glucosidase and β-xylosidase activity to hydrolyze oligosaccharides produced by fungal endoglucanase to provide the fungal culture with a carbon source for its growth.

Unlike fungi causing brown rot of wood, the synthesis of cellulases and xylanases in white rot fungi is subject to mechanisms of induction and catabolite suppression [11]. *P. chrysosporium* ATCC24725 is no exception. Available studies indicate that *P. chrysosporium* produces seven cellobiohydrolases, and five of them are efficiently secreted when the fungus is cultured in the presence of cellulose, cellooligosaccharides, or plant biomass substrates [36,37]. Cellotriose and cellotetraose clearly increased transcript levels of several cellulolytic genes in *P. chrysosporium*, while cellobiose and cellopentaose had little effect [36]. As shown in this study, *P. chrysosporium* ATCC24725 actively synthesizes cellulase and xylanase only in the presence of cellulose-containing growth substrates. Similar to *Fomes fomentarius*, *Panus lecometei*, *Trametes gibbosa*, *Trametes versicolor* [9], and *Schizophyllum commune* [13,38], cellulose was found to be the most suitable substrate for the production of cellulase and xylanase by *P. chrysosporium* ATCC24725, whereas CMC, a cellulose derivative with carboxymethyl groups, and xylan were poor substrates for the promotion of these enzymes’ secretion. However, a different pattern was observed in experiments with *Ganoderma applanatum*, where CMC and xylan were effective in inducing cellulase and xylanase production, although cellulose had the most significant induction effect on the secretion of these enzymes [39]. This fungus probably contains enzymes whose catalytic activity produces inducers that trigger transcription of cellulase and xylanase genes. Another distinctive feature of *G. applanatum* is the ability of the fungus to synthesize significant amounts of cellulase and xylanase when cultivated in a medium with xylose. In *P. chrysosporium* ATCC24725, as in other basidiomycetes listed above, hydrolase synthesis does not occur in the presence of easily metabolizable carbon sources due to catabolite repression, even if cellulose is available in the nutrient medium. Induction of enzyme synthesis occurs only after depletion of the easily metabolizable carbon source. Moreover, based on the findings of this study, it can be inferred that the production of endoglucanase and xylanase is co-regulated under all tested conditions. Similar results have been reported previously for other white-rot basidiomycetes [9,13,38]. These observations are consistent with the patterns of colonization and utilization of lignocellulosic materials by wood-destroying basidiomycetes, when the simultaneous secretion of a wide range of glycoside hydrolases facilitates rapid hydrolysis of polysaccharides available in the substrate.

## 5. Conclusions

Thus, this study identified a promising cellulase and xylanase producer, *P. chrysosporium* ATCC24725, which secretes high yields of target enzymes during submerged cultivation in an inexpensive nutrient medium. Cellulase and xylanase synthesis in this fungus is inducible in the presence of cellulose-containing materials, but crystalline cellulose is the best carbon source, promoting maximum yield of glycoside hydrolases. The addition of glucose or glycerol to a culture growing in a medium containing Avicel causes catabolite repression of cellulase and xylanase production in *P. chrysosporium* ATCC24725. However, enzyme synthesis resumes after depletion of the easily metabolizable carbon source from the medium. The fermentation process can be easily scaled up from flasks to a fermenter without reducing the productivity of the fungal culture. Moreover, CMCase and xylanase from *P. chrysosporium* ATCC24725 exhibit catalytic activity and stability over a wide range of pH values and temperatures, and they show promising potential for use when performing hydrolysis of plant residues to produce fermentable sugars.

## Figures and Tables

**Table 1:** Cellulase and xylanase activities of wood-rotting basidiomycetes in submerged fermentation of crystalline cellulose.

Fungi	Final pH	CMCase (U/mL)	FPA (U/mL)	Xylanase (U/mL)
*C. comatus* BCC801	8.3 ± 0.1 ^b^	3.0 ± 0.4^7^ *^e^	0.17 ± 0.02^4 d^	3.6 ± 0.3^4 e^
*F. fomentarius* BCC38	6.2 ± 0.1 ^f^	4.2 ± 0.6^10 de^	0.83 ± 0.10^10 b^	7.5 ± 0.8^10 de^
*I. lacteus* BCC104	6.8 ± 0.1 ^e^	47.5 ± 6.2^10 b^	2.31 ± 0.18^14 a^	53.7 ± 6.3^14 b^
*P. lecomtei* BCC903	6.3 ± 0.1 ^f^	6.8 ± 0.5^10 de^	0.52 ± 0.06^10 c^	8.1 ± 0.9^10 de^
*P. chrysosporium* ATCC24725	7.3 ± 0.1 ^c^	71.7 ± 9.8^10 a^	2.19 ± 0.20^14 a^	75.9 ± 9.1^10 a^
*P. betulinus* BCC520	8.5 ± 0.1 ^a^	0.2 ± 0^10 e^	0 ^d^	0.8 ± 0.1^4 e^
*P. ostreatus* BCC518	8.2 ± 0.1 ^b^	1.8 ± 0.3^10 e^	0.16 ± 0.02^7 d^	3.1 ± 0.3^10 e^
*T. gibbosa* BCC22	6.8 ± 0.1 ^e^	26.0 ± 3.1^10 c^	0.95 ± 0.08^7 b^	29.0 ± 3.7^10 c^
*T. ochracea* BCC76	7.1 ± 0.1 ^d^	11.3 ± 1.5^10 d^	0.39 ± 0.02^7 c^	11.9 ± 1.5^7 d^

* The numbers indicate the day of peak activity. Values sharing a common letter within the column are not significantly different at *p* < 0.05.

**Table 2:** Effect of different growth substrates on the cellulase and xylanase activities of selected fungi.

Growth Substrate	Final pH	CMCase (U/mL)	FPA (U/mL)	Xylanase (U/mL)
*P. chrysosporium* ATCC24725				
Avicel	7.2 ± 0.1 ^b^	72.1 ± 6.4^10^ *^a^	2.41 ± 0.16^10 a^	84.6 ± 7.2^7 a^
Tangerine pomace	7.6 ± 0.1 ^a^	20.3 ± 2.8^10 bc^	0.51 ± 0.07^4 b^	21.4 ± 3.3^10 b^
Vinasse from corn	6.7 ± 0.1 ^c^	28.3 ± 3.5^14 b^	0.43 ± 0.05^7 b^	28.8 ± 4.0^10 b^
Wheat bran	7.6 ± 0.1 ^a^	11.2 ± 1.0^10 c^	0.49 ± 0.06^10 b^	19.2 ± 2.7^14 b^
Wheat straw	6.7 ± 0.1 ^c^	17.8 ± 2.0^10 c^	0.48 ± 0.05^10 b^	23.9 ± 2.8^10 b^
*T. gibbosa* BCC22				
Avicel	6.9 ± 0.1 ^b^	23.5 ± 2.0^10 a^	0.85 ± 0.07^7 a^	26.8 ± 2.3^10 a^
Tangerine pomace	7.5 ± 0.1 ^a^	16.6 ± 1.5^10 b^	0.74 ± 0.08^7 ab^	2.8 ± 0.2^10 c^
Vinasse from corn	6.2 ± 0.1 ^d^	14.0 ± 1.5^14 bc^	0.41 ± 0.05^7 c^	6.0 ± 0.4^10 b^
Wheat bran	6.5 ± 0.1 ^c^	11.8 ± 1.4^10 c^	0.59 ± 0.05^4 b^	2.9 ± 0.3^10 c^
Wheat straw	6.6 ± 0.1 ^c^	13.4 ± 1.0^10 bc^	0.76 ± 0.07^4 ab^	4.3 ± 0.3^10 bc^
*T. ochracea* BCC76				
Avicel	7.2 ± 0.1 ^a^	10.4 ± 1.2^10 a^	0.45 ± 0.06^7 a^	13.4 ± 1.2^7 bc^
Tangerine pomace	7.0 ± 0.1 ^a^	10.2 ± 0.8^10 a^	0.25 ± 0.03^4 b^	39.5 ± 5.0^10 a^
Vinasse from corn	6.7 ± 0.1 ^b^	5.3 ± 0.6^14 b^	0.26 ± 0.04^7 b^	14.7 ± 2.0^10 b^
Wheat bran	6.3 ± 0.1 ^c^	4.9 ± 0.6^14 b^	0.28 ± 0.03^4 b^	7.2 ± 0.8^14 c^
Wheat straw	6.2 ± 0.1 ^c^	9.7 ± 1.2^10 a^	0.24 ± 0.03^7 b^	13.7 ± 2.0^10 bc^

* The numbers indicate the day of peak activity. Values sharing a common letter within the column are not significantly different at *p* < 0.05.

**Table 3:** Cellulase and xylanase activities of *P. chrysosporium* ATCC24725 in dependence on polysaccharide concentration.

Polysaccharide Concentration (%)	Final pH	CMCase (U/mL)	FPA (U/mL)	Xylanase (U/mL)
0 (control)	6.4 ± 0.1 ^de^	Traces	0 ^c^	0.2 ± 0^10 e^
	Avicel			
0.5	6.7 ± 0.1 ^bc^	27.5 ± 3.1^10^ *^c^	2.3 ± 0.2^14 b^	30.5 ± 4.1^10 b^
1.0	6.5 ± 0.1 ^cd^	69.3 ± 7.3^10 b^	2.7 ± 0.3^14 b^	83.7 ± 6.7^10 a^
2.0	6.2 ± 0.1 ^c^	87.9 ± 11.6^14 a^	3.3 ± 0.4^14 a^	90.8 ± 8.1^14 a^
	Carboxymethyl cellulose			
0.5	6.3 ± 0.1 ^de^	2.7 ± 0.3^4 d^	0.2 ± 0.01^7 c^	5.5 ± 0.4^7 cde^
1.0	7.0 ± 0.1 ^a^	4.1 ± 0.3^7 d^	0.2 ± 0.02^10 c^	11.0 ± 1.3^7 cd^
2.0	6.8 ± 0.1 ^ab^	4.8 ± 0.5^7 d^	0.3 ± 0.03^7 c^	13.8 ± 1.6^7 c^
	Xylan			
0.5	6.7 ± 0.1 ^bc^	0.2 ± 0.02^7 d^	0.1 ± 0.01^4 c^	0.8 ± 0.1^7 de^
1.0	6.5 ± 0.1 ^cd^	0.3 ± 0.02^7 d^	0.1 ± 0.02^4 c^	1.0 ± 0.1^4 de^
2.0	6.4 ± 0.1 ^de^	0.3 ± 0.03^7 d^	0.2 ± 0.02^7 c^	1.2 ± 0.2^7 de^

* The numbers indicate the day of peak activity. Values sharing a common letter within the column are not significantly different at *p* < 0.05.

**Table 4:** Pretreated wheat straw saccharification by *P. chrysosporium* ATCC24725 and commercial cellulases.

Enzyme Load	Reducing Sugars (mg/mL)	
	Duration of Hydrolysis	
	24 h	48 h
*P. chrysosporium* ATCC 24725 enzyme, 10 FPU/g	5.3 ± 0.1 ^b^	6.7 ± 0.1 ^b^
*P. chrysosporium* ATCC 24725 enzyme, 20 FPU/g	6.5 ± 0.1 ^a^	7.8 ± 0.1 ^a^
Accellerase 1500, 10 FPU/g	5.0 ± 0.1 ^c^	6.4 ± 0.1 ^c^
Accellerase 1500, 20 FPU/g	6.4 ± 0.1 ^a^	7.6 ± 0.1 ^a^

Values are presented as mean ± SD (*n* = 3). Values sharing a common letter within the column are not significantly different at *p* < 0.05.

## Data Availability

The original contributions presented in this study are included in the article. Further inquiries can be directed to the corresponding author.
